# Can the use of digital algorithms improve quality care? An example from Afghanistan

**DOI:** 10.1371/journal.pone.0207233

**Published:** 2018-11-26

**Authors:** Andrea Bernasconi, François Crabbé, Martin Raab, Rodolfo Rossi

**Affiliations:** 1 Swiss TPH, Basel, Switzerland; 2 University of Basel, Basel, Switzerland; 3 PHC programs, International Committee of the Red Cross, Genève, Switzerland; The University of Warwick, UNITED KINGDOM

## Abstract

**Background:**

Quality of care is a difficult parameter to measure. With the introduction of digital algorithms based on the Integrated Management of Childhood Illness (IMCI), we are interested to understand if the adherence to the guidelines improved for a better quality of care for children under 5 years old.

**Methods:**

More than one year after the introduction of digital algorithms, we carried out two cross sectional studies to assess the improvements in comparison with the situation prior to the implementation of the project, in two Basic Health Centres in Kabul province. One survey was carried out inside the consultation room and was based on the direct observation of 181 consultations of children aged 2 months to 5 years old, using a checklist completed by a senior physicians. The second survey queried 181 caretakers of children outside the health facility for their opinion about the consultation carried out through the tablet and prescriptions and medications given.

**Results:**

We measured the quality of care as adherence to the IMCI’s guidelines. The study evaluated the quality of the physical examination and the therapies prescribed with a special attention to antibiotic prescription. We noticed a dramatic improvement (p<0.05) of several indicators following the introduction of digital algorithms. The baseline physical examination was appropriate only for 23.8% [IC% 19.9–28.1] of the patients, 34.5% [IC% 30.0–39.2] received a correct treatment and 86.1% [IC% 82.4–89.2] received at least one antibiotic. With the introduction of digital algorithms, these indicators statistically improved respectively to 84.0% [IC% 77.9–88.6], >85% and less than 30%.

**Conclusions:**

Our findings suggest that digital algorithms improve quality of care by applying the guidelines more effectively. Our experience should encourage to test this tool in different settings and to scale up its use at province/state level.

## Introduction

In 1999 the Institute of Medicine (IoM) alerted the healthcare industry in the US about the lack of consistency in the delivery of quality care to the American people [1]. Defining quality of care is a complex question that has along and animated discussion. There are different conceptual approaches and measurement techniques to defining this. Furthermore, the concept spans on two levels, from the level of the individual patient to the level of the health system [2, 3] and the distinction between quality and performance is often unclear [2].

When asked about quality of care, patients, the direct beneficiaries, identify effective treatment and competent medical staff as the most important aspects of care [4]. Also, in low-middle income countries people living below the poverty line are inclined to bypass local services when perceived as having lower quality and prefer to access public services which are either geographically far or incur costs by addressing their health issues to the private sector [5]. Clinical skills were also highly valued by physicians, posing the perception of health providers and beneficiaries at the same level [6]. To ensure competency, the health care provided should be consistent with current professional knowledge [7] which, in turn, relies on evidence based medicine (EBM). “The effectiveness of clinical care depends on the effective application of knowledge-based care” stated Campbell [3]. Based on EBM, Clinical Practice Guidelines (CPGs) improve the quality of care by providing scientific standardized information that supports physicians in their clinical decision [8], especially these days that they are facing an exponential increase of medical knowledge [8, 9]. It is assumed that by developing CPGs on the base of good quality EBM, reflecting best practice and variation in practice, inappropriate care will be reduced [8, 10]. Despite this, usually physicians tend to overestimate their own ability to make a correct diagnosis without following guidelines and feel CPG too rigid [11, 12], the traditional paper-based guides too time consuming and they perceive that the use of paper tools may undermine the patients’ confidence in their skills [13, 14].

The Integrated Management of Childhood Illness (IMCI) can be viewed as a CPG proposed at the end of the ‘90’s by WHO/UNICEF to reduce child morbidity and mortality in countries with poor health infrastructure. IMCI, through a simple and standardized clinical guidance, backs up health workers at Primary Health Care (PHC) level to better respond to the five main diseases affecting children under 5 (malaria, measles, diarrhoea, malnutrition and acute respiratory diseases) [15–17]. Today IMCI is implemented in more than 102 countries [18] and it is associated with a 15% reduction in child mortality [19]. Unfortunately the uptake of the strategy was variable and challenging for many health systems [20–22]. Even in countries with more resources, like South Africa, it was highlighted that the IMCI program was not able to fulfil all its potentiality due to poor adherence, omission of aspects of the consultation, lack of clarity about what constituted an IMCI consultation, and poor record keeping [18].

Taking into account the pitfalls of the IMCI’s programs, since 2015, ALMANACH (ALgorithm for the MANagement of Acute CHildhood illnesses) is under developing by the Swiss TPH. ALMANACH is an electronic and upgraded version of the IMCI available on android system [23, 24] and belongs to the group of applications called “Clinical Decision Support Systems” (CDSS) (ALMANACH is a CDSS based on predictive algorithms following a decision tree. CDSS applications analyse medical data to assist healthcare providers to make clinical decisions at the point of care in 2005 two systematic reviews concluded that CDSS improved practitioner performance in 68% (out of 70 studies [25] and in 64% of the cases (out of 97 studies) [26]. ALMANACH recommends, during the consultation, when to provide preventive services, which examinations to perform, when to refer, when to use a rapid diagnostic test, what treatment to prescribe and what dosage (automatically calculated on the basis of age and/or weight). The algorithms of ALMANACH were customized to the needs of the local healthcare workers and population, taking into account the resources locally present at PHC level in terms of drugs, staff competency, laboratory equipment and the epidemiological profile of the population because CDSS can help providers to apply knowledge more effectively and improve patient outcome if the system is tailored to the needs of the users and acceptance is continuously monitored [27, 28].

In partnership with the International Committee of the Red Cross (ICRC), ALMANACH was used in three Afghan Red Crescent Society (ARCS)’s Basic Health Centres (BHC) in Kabul province since May 2016. All the BHCs have a medical doctor in charge of consultations and almost 9,000 children from 2 months to 5 years old (8 936, male 53.8%, median age 18 months (IQR 10, 30)) were examined with the support of ALMANACH since the onset of the intervention. Users had minimal computer skills and no comparable project had been carried out in the area previously.

## Methodology

In our study we wanted to assess the improved quality of care introduced by ALMANACH as a degree of adherence to IMCI’s guidelines and EBM as it was been well established that better adherence improves the quality of clinical care provided to sick children [29].

In details, we compared the performance of healthcare workers (HWs) in two ARCS clinics after one year of using ALMANACH compared to the baseline prior the implementation.

To have a benchmark to better understand the outcomes of ALMANACH, a baseline survey was carried out before implementation to assess the performance level of clinical activities. Two hundreds paediatric consultations per health facility were observed by a senior Afghan doctor not linked to the project. The sample size assumed a difference in effect of 15%, a power of 0.8 and an error rate of 5%. The external evaluator recorded the child’s age and sex, the complaints, the symptoms asked and the physical examination performed by the healthcare provider, and, finally, the diagnosis and medication prescribed. Symptoms expressed by the patients, signs assessed and prescribed therapy were compared together with the diagnosis and the IMCI’s guidelines.

Since the deployment of the electronic device, programmatic data (number of consultations and diagnosis) were in real time routinely uploaded to District Health Information System 2 (DHIS 2) for the benefit of the program and health managers and to draft a monthly epidemiological bulletin to be shared with the healthcare workers in order to make them aware of their clinical performance.

Although these routine data were regularly collected, we were not sure if at the moment of the consultation, the HWs were less likely to adopt the guidance of the CDSS or more likely to use their own judgment and override recommendations they felt were not appropriate. Hence, in July 2017, more than one year since the implementation of ALMANACH, the performance of clinical activities was again assessed by two different surveys:

1. Consultation room survey (CRS): this survey had an observer compiling information about the consultation of children at the BHCs through using a checklist. This survey was completed inside the consultation room: recording patient assessment, physical examination, diagnosis and prescribed therapy were carefully observed and documented. To facilitate the comparison, the CRS used the same checklist of the baseline survey.
2. Caretaker survey (CTS): to minimize the observation bias this survey was conducted with children’s caretakers (parents or guardians) immediately after the consultation and without the presence of the healthcare provider. Caretakers were asked about the consultation using the tablet and key information were collected about prescription and treatment received.

There was no link between CRS and CTS: CRS and CTS documented management of different children.

To calculate the sample size for both surveys, we know, on the basis of the routine data collected in more than one year, that 25% of the children received at least one antibiotic (ATB). With a confidence level of 95%, we estimated that 180 consultations (90 consultations per health facility) would be enough to document the ATB prescription with a confidence interval of ± 4.5. This sample size does not allow to compare the previous baseline data with the new results by stratifying per each health facility but it is sufficient for global comparison. The surveys were carried out only in two health facilities as the third BHC was excluded due to security concerns preventing supervision visits since February 2017.

Data were digitally collected. For this purpose, an electronic version of the questionnaires was created (CommCare, Dimagi inc.). Data were then exported to Microsoft Excel (2016) and to STATA (StataCorp. 2013. *Stata Statistical Software*: *Release 13*. College Station, TX: StataCorp LP) for further analysis.

Through this analysis we compared the results of the surveys with the baseline in terms of ATB prescription and compliance with the IMCI. Results are displayed in graphs and tables as proportions or medians. Whenever necessary the chi square test (or z-test) was used to investigate the differences, in this case we considered a significant difference when the p value was <0.05.

Before proceeding to any survey, caretakers and health providers’ consent was taken. The surveys were conducted in the framework of the programmatic project assessment of ICRC and ARCS and they were supported by these two organizations and they were exempt for ethical approval.

## Results

The baseline survey was carried out from January to February 2016 and 404 consultations were directly observed. Marginally over half of the children were male (217, 53.7%) with a median age of 22 months (IQR 9, 36). During the implementation of ALMANACH between May 2016 and December 2017, a total of 6’343 children (95% of all the children attending the BHCs) were examined through ALMANACH for a total of 7’318 diagnoses (Table 1).

**Table 1:** Demographic and basic service delivery data at the baseline and after the implementation of ALMANACH.

	Baseline	Routine§	CRS	CTS
***Total children***	404	6343	181	181
***Total diagnosis***	443	7318	228	236
***Diagnosis per patient***	1.1	1.1	1.2	1.3
***Male (%)***	217 (53.7)	3421 (53.9)	91 (50.3)	94 (51.9)
***Age in month (Median*, *IQR)***	22 (9–36)	18 (10–30)	22 (11.5,34)	23.5 (12–36)

§ Data collected by routine through the tablet

### Preventive measures

Before the implementation of ALMANACH, as documented by the baseline survey, prevention and screening were not routinely performed. Administration of albendazole to children of 12 months or more was very low (5.2%, [IC% 3.1–8.5]) and IMCI’s danger signs were sporadically investigated (1.5% [IC% 0.6–3.2]). Only one-third (32.4% [IC% 26.5–38.9]) of the children between three and 18 months had their vaccination card checked. Very few children (3.2% [IC% 1.9–5.4]) were weighed. Screening for malnutrition and vitamin A supplementation were never performed.

With the use of ALMANACH almost all children were weighed (97.8% [IC% 94.5–99.0] at CTS survey, 100% [IC% 97.9–100.0] at CRS and deworming and vitamin A supplementation increased respectively to 95.1% [IC% 89.8–97.7] and to 92.5% [IC% 87.3–95.7] for the CRS and to 94.2% [IC% 89.0–97.0] and 90.8% [IC% 85.5–94.4] for the CTS. The vaccination status was checked in almost all children (100% [IC% 95.6–100.0] for CRS and 94.3% [IC% 87.4–97.5] for CTS and 23.1% [IC% 17.3–30.2] were screened for malnutrition (Table 2).

**Table 2:** Prevention measures in place before and after the implementation of ALMANACH.

	BASELINE	ROUTINE	CRS	CTS
***Danger signs§checked (at least one) # (%)***	6 (1.5)	N/A	75 (41.4)*	N/A
**Tot. 2–60 months # (%)**	404 (100.0)		181 (100.0)	
***Weighed # (%)***	13 (3.2)	6324 (99.7%)	181 (100.0)*	177 (97.8)*
**Tot. 2–60 months # (%)**	404 (100.0)	6343 (100.0)	181 (100.0)	181 (100.0)
***MUAC measured # (%)***	0 (0.0)	N/A	37 (23.1)*	N/A
**Tot. ≥ 6–60 months**	352 (87.1)		160 (88.4)	
***Malnourishment detected # (%)***	0 (0.0)	132 (2.1)	5 (2.7)*	0 (0.0)
** **	404 (100.0)	6343 (100.0)	181 (100.0)	181 (100.0)
***Albendazole received # (%)***	14 (5.2)	1‘792 (31.3%)	117 (95.1)*	130 (94.2)*
**Tot. ≥ 12–60 months**	270 (66.9)	5‘723 (90.2%)	123 (67.9)	138 (76.2)
***Vitamin A received # (%)***	0 (0)	1‘900 (41.9%)	148 (92.5)*	149 (90.8)*
**Tot. ≥ 6–60 months**	352 (87.1)	4‘538 (71.5%)	160 (88.4)	164 (90.6)
***Vaccination status checked # (%)***	70 (32.4)	N/A	92 (100.0)*	83 (94.3)*
**Tot. ≥ 3–23 months**	216 (53.5)	N/A	92 (50.8)	88 (48.6)

*significant (p < .05) in comparison to the baseline survey

§ IMCI’s Danger signs include: convulsion currently or the recent past, unconsciousness/lethargy, vomit everything, inability to drink or to be breastfed

### Diagnoses

Based on the routine data over 16 months, respiratory infections counted for more than half (55.7%) of the diagnoses, followed by gastrointestinal infections (26.2%) and sore throat (10.7%) (Table 3).

**Table 3:** Diagnosis at the baseline, CRS and CTS and as reported by the routine data.

Diagnosis	Baseline		Routine		CRS		CTS	
	#	%	#	%	#	%	#	%
**Danger sign**	0	0.0	6	0.1	0	0.0	0	0.0
**Malnutrition**	0	0.0	132	1.8	5	2.2*	0	0.0
**Respiratory diseases**	261	58.9	4078	55.7	90	39.5*	98	41.5*
**Gastrointestinal diseases**	30	6.8	1915	26.2	68	29.8*	67	28.4*
**Sore throat**	77	17.4	784	10.7	15	6.6*	29	12.3
**Infectious diseases**	1	0.2	110	1.5	12	5.3*	7	3.0*
**Ear diseases**	17	3.8	180	2.5	3	1.3	1	0.4*
**Skin diseases**	22	5.0	89	1.2*	9	3.9	12	5.1
**Anaemia**	2	0.5	24	0.3	1	0.4	0	0.0*
**Parasitosis**	19	4.3	0	0.0	0	0.0*	0	0.0*
**Other**	14	3.2	0	0.0	6	2.6	14	5.9
**Healthy**	0	0.0	0	0.0	19	8.3*	7	3.0*
**Total**	**443**	**100.0**	**7318**	100.0	**228**	100.0	**235**	99.6

*significant (p < .05) in comparison to the baseline survey

Most diseases (83.8%) had no bacterial origin: upper respiratory tract infection (URTI) and viral infections accounted for 83.7% of the respiratory diseases; acute watery diarrhoea (AWD) accounted for 92.3% of the gastrointestinal infections. Sore throat’s bacterial aetiology can hardly be distinguished from the viral one only on basis of the clinical examination. ALMANACH considered the possibility of group A streptococcal (GAS) sore throat in any febrile child above 2 years old, without evidence of symptoms of viral infection (cough, rhinorrhoea, coryza or conjunctivitis). Based on this definition, 10.7% of sore throat cases were considered bacterial. In total, on the basis of the data collected over one year of activities, 1’187 cases out of the 7’318 (16.2%) warranted ATB therapy.

As the study was conducted during the summer season, the CRS and CTS surveys identified fewer respiratory diseases but more gastrointestinal diseases. For the first time some patients (3.0% CTS, 8.3% CTS), were considered healthy and were not prescribed any therapy. As a result, more systematic deworming (albendazole supplementation), parasitosis was no more reported.

### Physical examination

The review of clinical case management documented by the baseline study showed that only 23.8% [IC% 19.9–28.1] of patients underwent a proper physical examination in compliance with the IMCI protocol. Temperature was not measured in any febrile child. The presence of fever with cough/difficult breathing should be a trigger to check for the respiratory rate (RR) in order to exclude pneumonia. At the baseline, only 88 (40.5%) of the 217 symptomatic children had their RR checked. Moreover, the rate had been kept into account in only six of the 39 diagnosed cases of pneumonia (chest in drawing, other important sign for pneumonia, was not searched in any of the cases). We could assume that 84.6% of the cases of pneumonia at the baseline had been diagnosed without any substantial medical evidence or, at best, only by auscultation, which is neither objective nor reliable, especially when performed by poorly trained healthcare providers [30, 31]. All cases of sore throat (85) were treated as GAS infections despite only three met the case definition of GAS. Despite AWD is relatively easy to diagnose, the dehydration status was assessed in only one third of the cases (36.7%). In contrast, the CRS results showed that, with the introduction of ALMANACH, 84.0% [IC% 77.9–88.6] of children underwent a proper physical examination (p < .05): temperature was measured in 74.1% of the febrile children (p < .05), RR was counted in 52.4% of children presenting with cough and fever and the 88.8% (p < .05) of children with AWD were checked for dehydration.

### Treatment

At the baseline, one-third of the patients (34.5% [IC% 30.0–39.2]) received a therapy in line with the diagnosis and 23.9% of these treatments did not comply with IMCI recommendations. Antibiotics over prescription was the main cause of deviation from standard protocols: all cases of URTI were prescribed ATB, up to 49.2% of viral infections and 46.4% of AWD were treated with ATB. Almost nine children out of ten received an ATB at the baseline (86.1%, [IC% 82.4–89.2]). The remaining cases of incorrect therapy were mainly AWD cases (22, 78.6% of all AWD cases), and failed to receive the complementary zinc medication. Following the introduction of ALMANACH the percentage of children receiving a proper treatment increased drastically (98.8% [IC% 95.2–99.4] for CRS and 87.3% [IC% 81.6–91.4] for CTS) as did the percentage of treatments in line with the IMCI guidelines (92.8% CRS, 83.9% CTS). As a consequence of receiving a proper treatment, the percentage of children treated with one or more antibiotics dropped to 11.6% [IC% 7.8–17.1] (CRS) and to 31.5% [IC% 25.2–38.6] (CTS) (Table 4). Moreover, most of the prescribed antibiotics were recommended by IMCI (100% in CRS, 96.6% in CTS) against the 72,8% prescribed at the baseline, often, at the wrong dosage (p < .05).

**Table 4:** Global antibiotics prescription at the baseline, in one year of project and at the CRS and CTS.

Source of the data	Patient receiving at least one ATB	Total patient	%	Percentage difference in relation to the baseline	Diseases in need of ATB therapy (%)
***Baseline***	348	404	86.1		14.0
***Routine***	1193	6343	18.8*	-78.1 [IC% -68.3,-95.6]	16.2
***CRS***	21	181	11.6*	-86.5 [IC% -77.7,-92.2]	7.7
***CTS***	57	181	31.5*	-63.4 [IC% -52.9,-72.3]	12.7

*significant (p < .05) in comparison to the baseline survey

Although the percentages of diagnoses in need of ATB did not significantly change from the baseline (14.0%), the routine data (16.2%) collected and the CTS (12.7%) (only the CRS present a value significantly lower (7.7%)), healthcare providers showed the tendency to overprescribe ATB in presence of specific diseases or symptoms like sore throat, infection diseases and gastrointestinal disorders. The medical supply to the BHCs did not change in the last 2 years.

Clinical differentiation between a viral and bacterial aetiology of sore throat, with no support of laboratory tests is challenging; healthcare providers had the tendency to treat viral diseases, such as chickenpox and measles, with ATB; many cases of AWD were treated with metronidazole even without any evidence of blood in the stools. In conclusion, when counting all the diseases, we can conclude that 55.3% of baseline diagnoses received unwarranted ATB therapy, contrasting with 8.3% at CRS and 12.3% at CTS (Table 5).

**Table 5:** ATB overprescription (%) stratified by disease groups.

	Baseline	CRS	CTS
**Malnutrition**	0	-20*	0
**Respiratory diseases**	56.4	4.4	2.1
**Gastrointestinal diseases**	66.6	0	15.0
**Sore throat**	93.5	6.6	41.4
**Infectious diseases**	100	8.3	42.8
**Ear diseases**	-41.2	0	0
**Skin diseases**	36.4	0	0
**Anaemia**	50.0	0	0
**Parasitosis**	21.1	0	0
**Other**	-7.1	0	7.1
**Total**	55.3	8.3	12.3

* Minus sign in front of the percentage indicates diagnoses in need of ATB but treated without

(ie SAM cases who did not receive amoxicilline).

Comparing the baseline with the routine data in winter time (the baseline was carried out from January to February 2016), despite the percentage of diseases requiring antibiotic prescription was twice as high (34.0% vs 14.9%), the actual antibiotic prescription was halved (85.9% vs 38.1%) [24].

Regarding the therapies prescribed, the CRS found that home remedies, usually related with diet, were recommended for 9.6% of the children (they were not present at the baseline) and almost all the drugs prescribed (98.8%) complied with the IMCI guidelines.

The CTS presented similar results: most of the drugs (83%) prescribed were available in the health facility. Few prescribed drugs (9.2%) were not currently in use by ALMANACH and their availability at the health facility was scarce (31.9% against the 87.7% of the drugs currently recommended by ALMANACH).

Among the prescribed drugs not recommended by ALMANACH (as not part of the therapeutic suggestion of the IMCI), the most common were metoclopramide, ibuprofen, clarithromycin and dexamethasone+chloramphenicol, citralka (di-sodium hydrogen citrate), calcium syrup and pizotifen.

At the CTS, almost all the consultations (96.7%) were carried out with the support of the tablet. Most of the caretakers who had a consultation with ALMANACH expressed their satisfaction (76.6%) and the remaining did not have a specific opinion but at the end, all were comfortable with the use of the tablet in the consultation room. The 96.0% and 97.7% of the caretakers confirmed, respectively, that with ALMANACH many more questions about the health of the child were asked and the physical examination was more careful.

## Discussion

The ALMANACH project in Afghanistan ended in December 2017 not because it failed but as a result of the outstanding insecurity situation. All the data collected in Afghanistan has formed the proof of concept for a further implementation in Nigeria (Adamawa State).

This experience showed an important contribution of ALMANACH to improve quality of care by applying the guidelines more effectively with a direct effect in improving the patient outcome. By ALMANACH, an improved comprehensive and holistic physical examination, including preventive screening and measures, and a better rationalization of the drug prescription with an important reduction in ATB prescription was observed for a better performance of the BHCs. This reduction in antibiotic prescription with ALMANACH has been also described in Africa as 29.7% [32] and 15.4% [33]. Unfortunately, because ALMANACH was implemented in only 3 BHCs we are hesitating to draw the same conclusion for the whole health system in the Kabul Province. A better overview of the impact of the tool on the health system on its globality and complexity will be formulated through the scaling up experience in Adamawa (Nigeria) where the use ALMANACH will be extended to more than 400 health facilities.

During our study in Afghanistan we cannot underestimate some biases. It was not possible to eliminate the seasonality and direct observation biases but we did not notice any common problems evidenced by other authors for e.g. like a certain resistance to use the tool from the physicians in fear of seeing his/her clinician autonomy reduced or his/her competence questioned [13]. The utilization rate of ALMANACH was always above 90%. The only quantifiable problem encountered is an increase in the consultation time that increased from 2–3 minutes to 8–10 minutes (maximum 15 minutes) but on the other hand this led to an improvement inpatient care. Through using the paper-based algorithms it was reported the time for consultation increased from 20 minutes to an hour for a single consultation [18].

Insufficient knowledge and skills are considered the main causes of the poor performance of health workers in low income countries [34, 35] triggering major investment in training. To fill this gap and at the same time to improve quality of care, different strategies have been tested in the past: clinical guidelines, flowcharts, checklists, algorithms, etc … [36, 37, 38] CDSS is the latest and more technological advanced solution to this problem but they cannot work without additional and supportive interventions. ALMANACH can easily guarantee easy access to guidelines but its chance of success for an optimal quality of care relies on the quality of work environment in terms of availability of drugs, equipment, laboratory support and referral system. We deem that the major constraint for a full implementation of CDSS in low-income countries is the level of motivation showed by the users, as already suggested also by other authors [39, 40], especially in a context that works with a no incentive policy, in the long term HWs could be tired to work all the time with the usual algorithms. During our experience, we tried to trigger continuously the interest of the HWs by involving them in the development of the tool (workshop to collect ideas to adapt algorithms), by offering the chance to improve their professional advancement by learning how to use a CDSS, by guarantying access to EBM, by stimulating their management capacity through the access (via DHIS 2 dashboard installed in the tablet HWs can check in real time their performance) to the epidemiological data related to their BHCs and, finally, by reassuring the ones working in professional isolation about their clinical decision. Together with HWs, engaging health-service providers, communities and service users is another key element to uphold the use of the tool.

We also trust that ALMANACH, by uploading in real time the data during the consultation, is a tremendous boost for the health managers and epidemiologists and reinforces a more scientific and systematic approach to the use of information concerning interventions on quality.

There are still few studies focusing on impact of CDSS especially in relation with clinical outcomes [26, 41, 42]. We hope our experience could encourage other researchers to study the impact of CDSS on the quality of the medical decision taken in a moment when an increasing number of projects start to show promising results despite fragile infrastructure [43–46]. We believe that the development of CDSS software and the use of digital algorithms on EBM can make a dramatic difference not only in rural health facilities, which suffer from shortage of staff, training and where health providers work in professional isolation but also in much more developed health systems where “as medical science and technology has advanced at a rapid pace, the health care delivery system has floundered in its ability to provide consistently high-quality care to all” [7].

## Supporting information

S1 Checklist (DOC)

S1 Data (ZIP)
