# Supplementary material for: Can the use of digital algorithms improve quality care? An example from Afghanistan
Source: PLoS One. 2018 Nov 26;13(11):e0207233. doi: 10.1371/journal.pone.0207233 (PMC6261034; doi:10.1371/journal.pone.0207233)
Supplement: S1 Checklist — (DOC) [file pone.0207233.s001.doc]

STROBE Statement—checklist of items that should be included in reports of observational studies

|  | Item No | Recommendation |
| --- | --- | --- |
| **Title and abstract** | 1 | (*a*) Indicate the study’s design with a commonly used term in the title or the abstract  We used a title that can attract immediately the attention. The title focuses on quality of care, an important and discussed issue in Public Health |
|  |  | (*b*) Provide in the abstract an informative and balanced summary of what was done and what was found  Done |
| Introduction | | |
| Background/rationale | 2 | Explain the scientific background and rationale for the investigation being reported  We explain the importance of guidelines, evidence-based medicine and CDSS to achieve an improvement in quality of care. |
| Objectives | 3 | State specific objectives, including any prespecified hypotheses  Objective of our study is to understand if the introduction of a CDSS has improved the quality of care after one year since its implementation |
| Methods | | |
| Study design | 4 | Present key elements of study design early in the paper  We performed two cross sectional studies:  1.Consultation room survey (CRS): this survey had an observer compiling information about the consultation of children at the BHCs through a checklist. This survey was held inside the consultation room: patient assessment, physical examination, diagnosis and prescribed therapy were carefully observed and recorded.  2.Caretaker survey (CTS): this survey was conducted with children’s caretakers (parents or guardians) immediately after the consultation and without the presence of the healthcare provider (to minimize the observation bias). Caretakers were asked about the consultation through the tablet and key information about prescription and treatment received were gathered.  There was no link between CRS and CTS: CRS and CTS documented management of different children. |
| Setting | 5 | Describe the setting, locations, and relevant dates, including periods of recruitment, exposure, follow-up, and data collection  The surveys were carried out in July 2017 in two Basic Health Facilities (Primary Health Care) implementing digital algorithms in Kabul province. |
| Participants | 6 | All the children from two months to five years were invited to participate in the survey upon consensus of their caretakers |
|  |  |  |
| Variables | 7 | We consider as variables  Number of children weighed and receiving albendazole and vitamin A as prevention  Number of children receiving a proper physical examination as suggested by IMCI  Number of children receiving a proper therapy matching the diagnosis  Number of children receiving at least one antibiotic |
| Data sources/ measurement | 8* | Direct observation of the consultation and interviews with the caretaker outside the health facilities were the only source of data for our study |
| Bias | 9 | We consider two important biases:  Baseline and assessment surveys were carried out in different season  We should accept an important observation bias during the direct observation of the consultation at the health facilities |
| Study size | 10 | Explain how the study size was arrived at  On the base of the routine data collected, 25% of the children receive at least one antibiotic (ATB). With a confidence level of 95%, we estimated that 180 consultations (90 consultations per health facility) would be enough to document the ATB prescription with a confidence interval of ± 4.5. This sample size does not allow to compare the previous baseline data with the new results by stratifying per each health facility but it is sufficient for global comparison. |
| Quantitative variables | 11 | Explain how quantitative variables were handled in the analyses. If applicable, describe which groupings were chosen and why  Data collected by the surveys were directly entered into tablets for further analyses. For this purpose, an electronic version of the questionnaires was created (CommCare©, Dimagi inc.). Data were then exported to Microsoft Excel (2016) and to STATA (StataCorp. 2013. Stata Statistical Software: Release 13. College Station, TX: StataCorp LP) for further analysis |
| Statistical methods | 12 | Results are displayed in graphs and tables as proportions or medians. Whenever necessary the chi square test was used to investigate the differences, in this case we considered a significant difference when the p value was <0.05. |

| Results | | |
| --- | --- | --- |
| Participants | 13* | (a) Report numbers of individuals at each stage of study—eg numbers potentially eligible, examined for eligibility, confirmed eligible, included in the study, completing follow-up, and analysed  All the children from two months to five years old attending the health facilities implementing digital algorithms were invited to participate in the survey upon consensus of their caretakers. At least 90 children per health facilities were enrolled in the study |
|  |  | (b) Give reasons for non-participation at each stage  Clearly request to not participate into the survey |
|  |  | (c) Consider use of a flow diagram |
| Descriptive data | 14* | (a) Give characteristics of study participants (eg demographic, clinical, social) and information on exposures and potential confounders  All the children from two months to five years old attending the health facilities implementing digital algorithms during the time of the surveys |
|  |  | (b) Indicate number of participants with missing data for each variable of interest  No one |
|  |  | (c) *Cohort study*—Summarise follow-up time (eg, average and total amount) |
| Outcome data | 15* | *Cohort study*—Report numbers of outcome events or summary measures over time |
|  |  | *Case-control study—*Report numbers in each exposure category, or summary measures of exposure |
|  |  | *Cross-sectional study—*Report numbers of outcome events or summary measures  Main outcomes we assessed were:  Percentage of children weighed and receiving albendazole and vitamin A as prevention  Percentage of children receiving a proper physical examination as suggested by IMCI  Percentage of children receiving a proper therapy matching the diagnosis  Percentage of children receiving at least one antibiotic |
| Main results | 16 | 1. Give unadjusted estimates and, if applicable, confounder-adjusted estimates and their precision (eg, 95% confidence interval). Make clear which confounders were adjusted for and why they were included   Percentage of children weighed: 97.8% [IC% 94.5-99.0] at caretaker survey (CTS), 100% [IC% 97.9-100.0] at consultation room survey (CRS)  Percentage of children receiving albendazole A as prevention: 95.1% [IC% 89.8-97.7] (CRS); 90.8% [IC% 85.5-94.4] (CTS)  Percentage of children receiving vitamin A as prevention: 92.5% [IC% 87.3-95.7] CRS); 90.8% [IC% 85.5-94.4] (CTS)  Percentage of children receiving a proper physical examination as suggested by IMCI: 84.0% [IC% 77.9-88.6] (CRS)  Percentage of children receiving a proper therapy matching the diagnosis: 98.8% [IC% 95.2-99.4] (CRS); 87.3% [IC% 81.6-91.4] (CTS)  Percentage of children receiving at least one antibiotic: 11.6% [IC% 7.8-17.1] (CRS); 31.5% [IC% 25.2-38.6] (CTS) |
|  |  | (*b*) Report category boundaries when continuous variables were categorized |
|  |  | (*c*) If relevant, consider translating estimates of relative risk into absolute risk for a meaningful time period |
| Other analyses | 17 | Report other analyses done—eg analyses of subgroups and interactions, and sensitivity analyses  No other analyses done |
| Discussion | | |
| Key results | 18 | Summarise key results with reference to study objectives  The implementation of digital algorithms dramatically improves the quality of care of the patients by assuring adherence to the protocol in terms of preventive measures, physical examination and therapy with a considerable decrease in antibiotic prescription |
| Limitations | 19 | Discuss limitations of the study, taking into account sources of potential bias or imprecision. Discuss both direction and magnitude of any potential bias  We consider two important biases:  Baseline and assessment surveys were carried out in different season  We should accept an important observation bias during the direct observation of the consultation at the health facilities  Because the project was implemented in only 3 BHCs we are hesitating to draw the same conclusion for the whole health system in the Kabul Province or other different settings |
| Interpretation | 20 | Give a cautious overall interpretation of results considering objectives, limitations, multiplicity of analyses, results from similar studies, and other relevant evidence |
| Generalisability | 21 | Discuss the generalisability (external validity) of the study results  Because the project was implemented in only 3 BHCs we are hesitating to draw the same conclusion for the whole health system in the Kabul Province or other different settings but it is a proof that when digital algorithms are well designed, tailored on the health workers’ needs and resources and the health workers are working with a close support we can expect an important improvement in quality of care |
| Other information | | |
| Funding | 22 | Give the source of funding and the role of the funders for the present study and, if applicable, for the original study on which the present article is based  Surveys were carried out in the framework of the activities of Afghan Red Crescent Society and International Committee of the Red Cross |
